# Cancers Related to Immunodeficiencies: Update and Perspectives

**DOI:** 10.3389/fimmu.2016.00365

**Published:** 2016-09-20

**Authors:** Esmaeil Mortaz, Payam Tabarsi, Davod Mansouri, Adnan Khosravi, Johan Garssen, Aliakbar Velayati, Ian M. Adcock

**Affiliations:** ^1^Department of Immunology, Faculty of Medicine, Shahid Beheshti University of Medical Sciences, Tehran, Iran; ^2^Chronic Respiratory Research Center, National Research Institute of Tuberculosis and Lung Diseases (NRITLD), Shahid Beheshti University of Medical Sciences, Tehran, Iran; ^3^Division of Pharmacology, Faculty of Science, Utrecht Institute for Pharmaceutical Sciences, Utrecht University, Utrecht, Netherlands; ^4^Clinical Tuberculosis and Epidemiology Research Center, National Research Institute of Tuberculosis and Lung Diseases (NRITLD), Shahid Beheshti University of Medical Sciences, Tehran, Iran; ^5^Nutricia Research Centre for Specialized Nutrition, Utrecht, Netherlands; ^6^Mycobacteriology Research Center (MRC), National Research Institute of Tuberculosis and Lung Diseases (NRITLD), Shahid Beheshti University of Medical Sciences, Tehran, Iran; ^7^Cell and Molecular Biology Group, Airways Disease Section, Faculty of Medicine, National Heart and Lung Institute, Imperial College London, London, UK

**Keywords:** primary immunodeficiency, malignancy

## Abstract

The life span of patients with primary and secondary immunodeficiency is increasing due to recent improvements in therapeutic strategies. While the incidence of primary immunodeficiencies (PIDs) is 1:10,000 births, that of secondary immunodeficiencies are more common and are associated with posttransplantation immune dysfunction, with immunosuppressive medication for human immunodeficiency virus or with human T-cell lymphotropic virus infection. After infection, malignancy is the most prevalent cause of death in both children and adults with (PIDs). PIDs more often associated with cancer include common variable immunodeficiency (CVID), Wiskott–Aldrich syndrome, ataxia-telangiectasia, and severe combined immunodeficiency. This suggests that a protective immune response against both infectious non-self-(pathogens) and malignant self-challenges (cancer) exists. The increased incidence of cancer has been attributed to defective elimination of altered or “transformed” cells and/or defective immunity towards cancer cells. The concept of aberrant immune surveillance occurring in PIDs is supported by evidence in mice and from patients undergoing immunosuppression after transplantation. Here, we discuss the importance of PID defects in the development of malignancies and the current limitations associated with molecular pathogenesis of these diseases and emphasize the need for further knowledge of how specific mutations can modulate the immune system to alter immunosurveillance and thereby play a key role in the etiology of malignancies in PID patients.

## Introduction

The earliest evidence that individuals with primary immunodeficiencies (PIDs) develop cancer was reported in 1963 (1, 2). An increasing number of reports subsequently indicated that subjects with primary abnormalities of the immune system are at a high risk for developing cancer (3–6) and PID biology represents a rapidly developing area of clinical immunology. PIDs are inherited disorders of the immune system in which at least one, and often more, immune component is decreased, missing, or has an inappropriate function. PIDs comprise more than 130 different heterogeneous disorders that affect various aspects of immune development and function as well as the morphology of the immune system (7–10). PIDs are rare with a prevalence in the United States of 1:1200 live births (11), and the overall risk for children with PIDs of developing malignancy is estimated at 4–25% (12, 13). The type of malignancy that is seen is highly dependent on the precise PID, the age of the patient, and probably viral infection indicating that different pathogenic mechanisms may be implicated in each case (12).

As stated above, PIDs were originally described as rare, to only occur in infants and young children, and to be associated with severe clinical symptoms. However, advances in gene sequencing technologies, such as whole exome sequencing, have revealed that they are much more common than originally appreciated and are present in older children, adolescents, and adults, and can present with relatively mild clinical disease in some patients (14, 15).

PIDs are classified according to which component of the immune system is primarily involved. Defects in adaptive immune responses include antibody deficiency syndromes, combined immunodeficiencies (CIDs), and severe combined immunodeficiency (SCID) (16), whereas defects in innate immunity comprise disorders of phagocytes, and toll-like receptor (TLR)-mediated signaling and complement (17). All these forms of disease are characterized by increased susceptibility to recurrent infections and/or severe infections with the susceptibility to specific pathogens dependent upon the nature of the specific immune defect (18).

Immune dysregulation is present in some forms of PIDs, while other forms of PID are more complex and immunodeficiency represents only a part of the phenotype (immunodeficiency syndromes) (19, 20). The early detection of patients with PID is critically important as effective therapy is available for virtually all of the different disorders but is most beneficial when instituted before target organ damage has occurred (e.g., in the lung) by infection or by autoimmunity (21). Similarly, early recognition of primary immunodeficiency may lead to a precise genetic diagnosis, which in turn may be important to the family in planning their future reproductive options (22).

Genomic instability due to defective DNA repair processes and other unknown mechanisms in PID patients leads to an enhanced risk of cancer. We have reviewed here the etiology, pathogenesis, clinical, and laboratory features of each of the various categories of PIDs and highlighted their association with various malignancies.

## The Link between PIDs and Malignancies

Primary immunodeficiencies are occasionally referred to as inborn errors of immunity, inherited immunodeficiencies, heritable immune defects, or congenital immunodeficiencies. In all cases, it is explicit or implicit that the primary lesions are present in the germline, whether due to a *de novo* mutation or due to a mutation inherited from a parent carrying the mutation. It has also become evident that a normal cellular and clinical phenotype may be restored in some patients with PIDs when somatic mutations occur in the hematopoietic cell lineage (23, 24). As predicted by Burnet, there is an increased predisposition to malignancy in patients with severe primary immunodeficiency (1, 13, 25, 26).

PIDs are a heterogeneous group of rare disorders characterized by impaired humoral and/or cell-mediated immunity, in the absence of any recognized cause such as drug treatment or human immunodeficiency virus (HIV). The most common PID syndromes are common variable immunodeficiency (CVID) with a 25-year mortality rate of 24% mostly due to lymphoma (18%), chronic pulmonary disease (11%), and X-linked agammaglobulinemia (XLA), which accounts for between 80 and 90% of all cases (21, 27, 28). This compares with the survival rate of 92% (males) and 94% (females) within the general population (27). There is an enhanced incidence of several cancers including lymphoma and of stomach, breast, bladder, and cervical epithelial cancers in these patients with CVID associated with the presence of defective humoral immunity (12, 13, 25, 26). This highlights the importance of an effective immune response against infection in the prevention of oncogenesis.

### Common Variable Immune Deficiency

Common variable immune deficiency (CVID) is characterized by recurrent sino-pulmonary infections, autoimmune disorders, and granulomatous disease with an increased risk of malignancy (12- to 18-fold greater than in the general population) (29–34). CVID affects both children and adults, with an estimated prevalence of 1:25–50,000 (35, 36). The hallmark of CVID is hypogammaglobulinemia due to impaired B cell differentiation. CVID is heterogeneous, with most patients suffering mainly from infections, while others are particularly prone to non-infectious complications. These differences correlate with the particular B cell phenotype present in each patient (37).

An increased risk of malignancy particularly that of lymphoma and gastric cancer is associated with CVID (38–41). Indeed, malignancy is recognized as one of the five clinical CVID phenotypes proposed by Chapel and colleagues (38). In this classification, patients with polyclonal lymphadenopathy were shown to have fivefold increased risk of lymphoid malignancy (38). Lymphoid malignancy generally occurs late in disease and in patients with pre-existing polyclonal lymphocytic infiltration (38). However, lymphoma can appear in young pediatric CVID patients in the absence of previous polyclonal lymphadenopathy (42).

Lymphoma is one of the more severe complications of CVID, but the driver(s) of this increased risk are unclear, although it seems to be multifactorial with the interplay of genetics, immune dysregulation, and chronic infectious agents including non-oncogenic and oncogenic viruses such as Epstein–Barr virus (EBV) being important (43). This increase in infection, therefore, may be a critical driver of malignancy in patients with CVID. CVID-associated lymphomas are more likely to be of B cell origin with a predominance of non-Hodgkin lymphoma (NHL), and these usually occur in the fourth to seventh decades of life and are rarely seen in children. NHL is frequently extranodal and usually EBV-negative (43), and although the parotid gland, sinuses, orbital cavity, and stomach can be affected, the majority of cases affects the lung (44, 45).

## Hyper-IgE Syndrome (Job’s Syndrome)

Job’s syndrome is a complex CID disease that was first described in 1966 and named in reference to the Biblical character Job who was “smote with sore boils” (46). The description of the syndrome was refined after the realisation that in addition to the occurrence of recurrent boils, eczema, and pneumonias, these patients also had very high serum IgE levels (47). Two distinct entities have been recognized: the classical hyper-IgE syndrome, which is inherited in an autosomal-dominant pattern [autosomal-dominant hyper-IgE syndrome (AD-HIES)], and the autosomal recessive hyper-IgE syndrome. The vascular, skeletal, and connective tissue problems seen in AD-HIES reflect the multisystem status of the disorder (48, 49).

A mutation in the signal transducer and the activator of the transcription 3 (STAT3) gene has been identified in the majority of AD-HIES patients, which results in impaired Th17 cell differentiation and downregulation of antimicrobial responses (50–55). Chronic mucocutaneous candidiasis (CMC) patients with a dominant-negative STAT3 mutation present with a decreased number of central memory CD4+ and CD8+ lymphocytes and an increased number of naive T cells (56). Due to this loss of memory T cells, patients with HIES are predisposed to develop varicella zoster virus reactivation and prolonged EBV viremia. The impaired IL-17 + T cell differentiation and function is a plausible explanation for the susceptibility of HIES patients to CMC (51, 57). However, such patients are also susceptible to infections by pyogenic bacteria, especially *Staphylococcus aureus* causing recurrent skin and lung infections due to a failure of Th17 CD4+ cells to recruit neutrophils to the site of infection and to upregulate antimicrobial peptides (58).

Reduced levels of IL-17 are linked to mucocutaneous infections with *Candida albicans* in man (59–63) and to enhanced susceptibility to *Candida* and *Klebsiella* infections in mice (64, 65). Th17 cells are also important for IL-22 secretion, which is critical for beta-defensin production and subsequent protection against *S. aureus* infection in individuals with eczema (66). This suggests that the *S. aureus*-infected abscesses seen in AD-HIES are due to reduced beta-defensin production secondary to the lack of Th17 cells. Beta defensins are also expressed in the lung and might in part explain the susceptibility to pneumonias (67).

The reason why AD-HIES subjects have a very high serum IgE levels is unknown, although this has been linked to impaired IL-21 signaling (68). However, human B cells differ from those in mice in that IL-21 acts synergistically with IL-4 to increase IgE production in man (69), whereas IgE levels in serum are increased in IL-21R- and IL-21-deficient mice (68, 70). Therefore, the lack of IL-21 might be associated with less, not more, IgE in man. The combined effect of IL-4 and IL-21 on IgE secretion was also unrelated to alterations in expression of their receptors because neither IL-4 nor IL-21 enhanced the level of CD40L-induced IL-21R or IL-4R expression on B cells. It is possible that the increased IgE reflects a lack of suppression of IL-21 and/or IL-4 by IL-10 or IFNγ. It is noteworthy that this cardinal feature of AD-HIES has not yet been clearly explained.

A similar, but distinct, syndrome was reported in 2004, which was characterized by extremely elevated serum IgE levels, severe eczema, and recurrent bacterial and viral skin infections as well as by sino-pulmonary infections (71). In comparison to AD-HIES, these individuals lack the somatic features such as the characteristic faces, scoliosis, and the failure of baby teeth to exfoliate. In addition, although pneumonias occur in autosomal recessive hyper IgE syndrome (AR-HIES), pneumatoceles do not form.

Autosomal recessive hyper IgE syndrome has a much higher rate of coetaneous viral infections such as molluscum contagiosum, herpes simplex, and varicella infections. They also have frequent neurologic disease, ranging from facial paralysis to hemiplegia often due to CNS vasculitis. Mortality is high in younger patients with AR-HIES with sepsis being more frequent than in AD-HIES. Two other autosomal recessive forms of HIES are caused by mutations in the gene for tyrosine kinase 2 (Tyk2). Kilic et al. (72) described a patient with Tyk2 deficiency who displayed none of the other three cardinal features of HIES: atopic dermatitis and eczema, staphylococcal infections of the skin and lung, or high serum IgE concentrations; his highest recorded IgE concentration being 218 IU/mL at the age of 16 years. A diagnosis of Tyk2 deficiency should be contemplated in patients with BCG clinical disease, particularly in the presence of unusually severe herpes virus infection, even in the absence of cardinal features of HIES. This diagnosis may also apply to patients with other mycobacterial diseases, whether they are tuberculous or atypical, as well as those with infections caused by other intramacrophage pathogens, such as *Salmonella* and *Brucella*.

A related syndrome is seen in patients with defects in the gene dedicator of cytokinesis (DOCK) 8 (73–78). Patients with DOCK8 mutations have elevated IgE levels and the presence of allergy, eczema, cutaneous viruses, and malignancies (79). Contrary to its initial description as a form of hyper-IgE syndrome, DOCK8 deficiency can be regarded as a CID disease that features eczema and elevated IgE much like the Wiskott–Aldrich syndrome (WAS) (80). On the other hand, phosphoglucomutase 3 (PGM3) deficiency is not associated with cold abscesses, CMC, retained childhood dentition, and joint hypermobility that are seen very commonly in patients with STAT3 mutations. PGM3 deficiency can present with elevated IgE, atopic dermatitis, bronchiectasis, and scoliosis, which help to distinguish this disorder from the two best-known diseases with elevated IgE: STAT3 and DOCK8 deficiencies. Although PGM3-deficient patients occasionally have viral skin infections, they were not as prevalent as in DOCK8 deficiency. Primary neurocognitive deficits are seen in PGM3 deficiency but not in either STAT3 or DOCK8 deficiencies (81).

In the context of this review, HIES patients have a much higher risk of developing aggressive B cell lymphomas, which may be linked to abnormalities in STAT3/IL-21-dependent differentiation of B cells into plasma cells with the possible involvement of T follicular helper cells (82–84). B-cells are also important in the formation, progression, and metastases of many other cancers including lung cancer and melanoma (85, 86). Viral infection in AD-HIES and AR-HIES is associated with an increased risk of cancer over and above that seen with PIDs alone, suggesting that the inability of these patients to mount an efficient immune response to infection may allow pre-malignant cells to grow unchecked and develop into cancer. The role of Th17 and memory T-cells in this process needs to be better defined.

## Chronic Mucocutaneous Candidiasis and CMC Disease

Several PIDs typically characterized by CMC and impaired IL-17-mediated immunity have recently been identified (87). CMC in such patients may be part of a complex clinical phenotype exemplified by a dominant-negative STAT3 deficiency, interleukin (IL)-12Rβ1 and IL-12p40 deficiencies, and autoimmune polyendocrine syndrome type 1 (APS-1) (57, 88–90). In a subgroup of PIDs, a predisposition to superficial candidiasis may be the only, or at least the dominant, characteristic of the underlying genetic disorder [chronic mucocutaneous candidiasis disease (CMCD)] (91). Inborn errors of IL-17-mediated immunity lead to CMC in humans, and an autosomal-dominant mutation in IL-17F and autosomal recessive mutations in IL-17RA or IL17-RC predispose patients to complete CMC. In contrast, the heterozygous STAT1 mutations associated with CMC are gain-of-function (92–94).

*Candida albicans* is the most common species isolated from patients with CMC. Candidal infections develop and persist, usually beginning during infancy but sometimes during early adulthood. The fungus may cause mouth infections (thrush) and infections of the scalp, skin, and nails. Membranes lining the mouth, eyelids, digestive tract, and vagina may also be infected. In infants, the first symptoms are often difficult to treat thrush and diaper rash. The disorder may cause one or more nails to thicken, crack, and become discolored. A disfiguring rash may cover the face and scalp. The rash is crusted and thick and may ooze and when present on the scalp may cause hair to fall out (95).

Th17 cytokines are important for the prevention of infection with *Candida* colonizing the mucocutaneous surfaces (57, 61, 96). The differentiation, expansion, and maintenance of human IL-17-producing T cells is regulated by a set of cytokines including IL-1β, IL-6, IL-21, IL-23, and TGF-β along with the transcription factors STAT3, retinoic acid-related orphan receptor (ROR)-γt, and interferon regulatory factor 4 (IRF4) (48, 91, 97–100). However, alternative mechanisms to explain the unique vulnerability to infection of the skin, mucous membranes, and the lung in HIES patients may be required.

Chronic mucocutaneous candidiasis is associated with squamous cell cancers of the esophagus and of the oral cavity (95). This provides a link between the site of repeated or recurrent infection and a defect in the Th17 response. These cancers are also seen in patients with autosomal recessive autoimmune polyendocrinopathy syndrome type I (AR APS-I), which is caused by mutations in the protein autoimmune regulator (AIRE), who are not susceptible to any other infectious disease (91). The distinct mechanisms that underpin the increased incidence of esophagal cancer in CMC patients require further study (101).

## Familial Hemophagocytic Lymphohistiocytosis

The rare autosomal recessive disorder termed familial hemophagocytic lymphohistiocytosis (FHL) is due to defects in natural killer (NK) and cytotoxic T lymphocytes (CTLs), resulting in a multisystem inflammatory disease with persistent fever and hepatosplenomegaly associated with cytopenias and blood metabolic alterations. Type 2 FHL (FHL2) is linked to mutations in the PRF1 gene encoding human perforin, which represent 13–58% of the cases reported to date depending on the ethnicity of the patient (102–108). To date, more than 70 mutations in PRF1 have been described in FHL2. Although most of these are missense mutations, it is unclear how they affect protein expression and function (109–111). Studies of missense mutations from patient-derived cells and cell lines are limited by the infrequent occurrence of individuals with homozygous mutations.

Overall, there is a striking similarity between the biologic changes induced by proinflammatory cytokines and the clinical and laboratory findings in FHL (112). The proinflammatory cytokines soluble IL-2 receptor, IL-6, interferon-γ, and TNF-α are reported to be commonly elevated in FHL (112–114). More recent studies indicated elevated plasma levels of IL-12 and IL-10 in these patients. The former stimulates the production of Th1 cytokines, and the latter suppresses Th1 responses. In contrast, the levels of the Th2-stimulating cytokine IL-4 were not increased (115). It is reasonable to assume, therefore, that most of the symptoms, signs, and the laboratory alterations in FHL patients are mediated by proinflammatory cytokines.

Mutations in the PRF1 gene result in a reduced ability of immune cells to perform their essential immunosurveillance roles, particularly against spontaneous lymphomas, which accounts for the enhanced risk of lymphoma in FHL patients. Furthermore, a third of the patients with interleukin-10 receptor (IL-10R) deficiency, which results in abnormal Th1 responses and a loss of immunosurveillance capacity, develop B-cell lymphomas in the first decade of their life. The lymphomas uniformly contained amplifications of c-rel, activation of inflammatory nuclear factor κB (NF-κB)-induced target genes, and defective intratumoral CD81 T-cell tumor immunosurveillance (116).

## IgA Deficiency

Selective IgA deficiency is one of the most prevalent PID subtypes with an estimated prevalence of ~1:600 (117). Although selective IgA deficiency is asymptomatic in many patients, clinical manifestations include respiratory and GI infections, atopy, autoimmune diseases, and lymphoid and GI malignancies, which occur later in life (118). Since IgA deficiency and CVID share a common genetic basis, they may be considered as a spectrum ranging from the mild reductions in IgA levels to severe deficiencies of multiple antibodies in CVID (119). The progression from mild or asymptomatic selective IgA deficiency to CVID is possible and, if noted, should prompt initiation of Ig replacement therapy to prevent infectious and pulmonary complications (120). Patients with selective IgA deficiency have a high incidence of gastrointestinal cancers, highlighting the immunoprotective role of IgA against this type of malignancy.

Patients with X-linked immunodeficiency with hyper-IgM, caused by mutations in the CD40 ligand, have a high incidence of tumors of the pancreas and liver (121). Interestingly, a primary cutaneous marginal zone lymphoma (PCMZL) with the sequential development of nodal marginal zone lymphoma has been recently reported in a patient with selective immunoglobulin A deficiency (122). This is in addition to a previous report of a γ/δ type abdominal T-cell non-Hodgkin’s lymphoma in a patient with selective IgA deficiency (123).

## DNA Repair Disorders

A number of rare PIDs are the result of genetic defects in DNA repair that also lead to immune deficits and susceptibility to malignancy (124, 125).

## Ataxia-Telangiectasia

Ataxia-telangiectasia (AT) is an autosomal recessive disorder and is characterized by progressive cerebellar ataxia presenting in infancy, oculocutaneous telangiectasia, and dysarthria (126, 127). Cells from patients with AT have a reduced ability to activate cell cycle checkpoints, which is seen particularly following radiation exposure for example (γ-irradiation or radiomimetic agents) because of mutations in the AT mutated (ATM) gene. The ATM gene normally acts as a sensor of double-stranded DNA breakage (124), and the predisposition to leukemia is thought to be related to excessive production of DNA translocations (126).

Not surprisingly, therefore, AT has the highest risk for malignancy of any PID (126, 128), and the overall incidence of cancer among patients with AT is as high as 40%. Leukemias and lymphomas are particularly prevalent, appearing in AT patients with rates 70- to 500-fold and 200- to 750-fold, respectively, higher than in the general population (127). Interestingly, there is an increased risk of breast cancer among heterozygote AT carriers, who are otherwise generally healthy with the greatest risk in elderly AT patients who are smokers (129). The ability of immune checkpoint blockers to treat a number of different cancers also indicates a key role for defects in immune function being critical for the development of cancer.

## Nijmegen Breakage Syndrome

Nijmegen breakage syndrome (NBS) is a rare autosomal recessive disorder closely related to AT that, although it occurs worldwide, it has a greater prevalence in people of Central and Eastern European descent (130). The defective NBS gene (NBS1, nibrin, or p95) is a component within the same pathway as ATM and both proteins are part of a multi-subunit complex involved in correcting radiation-induced chromosomal aberrations (124, 131). Immune deficiency is generally severe and characterized by both humoral, such as agammaglobulinemia, IgA deficiency, and IgG2 and IgG4 deficiency, and cellular immunity, including lymphopenia, decreased CD31+ and CD41+ T helper cells, and a decreased CD41:CD81 T suppressor cell ratio (132). The physical features of NBS are typified by microcephaly, short stature, and “bird-like” faces (131, 132).

Not surprisingly, therefore, NBS exhibits many similarities to AT, including humoral and T-cell defects, radiosensitivity, and chromosomal instability, which are linked to immune dysfunction and to a high risk of malignancy.

## Wiskott–Aldrich Syndrome

Wiskott–Aldrich syndrome is a rare X-linked primary immunodeficiency characterized by microthrombocytopenia, eczema, recurrent infections, and an increased incidence of autoimmunity and malignancies. The disease is caused by mutations in the WAS gene, which is expressed only in hematopoietic cells (133). The overall incidence of cancer in WAS patients is unclear, although 18% of WAS subjects in a small study were reported as having malignant lymphoma (134). In all but one patient, the diagnosis of lymphoma was made ante-mortem and located predominantly in extranodal sites or within the brain. There was no involvement of peripheral lymph nodes (134). A higher incidence of NHL has also been described in WAS patients (135).

## Phagocyte Disorders

Severe congenital neutropenia (SCN) is a heterogeneous disorder due to the arrest of myelopoiesis maturation at the promyelocyte/myelocyte stage, resulting in neutropenia with systemic neutrophil blood counts <0.5 × 10^9^/L. Patients with SCN present with recurrent, severe infections during infancy and persistent neutropenia. A total of 50–60% of cases are associated with mutations in the elastase (ELA) 2 gene, and >50 unique mutations have been identified. Other common genetic defects associated with SCN include biallelic mutations in the hematopoietic cell-specific Lyn substrate 1 (HCLS1)-associated protein X-1 (HAX1) or in glucose-6-phosphatase, catalytic, 3 (G6PC3) genes. For most patients, daily treatment with granulocyte colony-stimulating factor (G-CSF) results in elevated blood neutrophil counts and reduces the risk of infection (136).

Heterozygous mutations in GATA2 resulting in GATA2 deficiency is a recently described disorder of hematopoiesis, lymphatics, and immunity. GATA2 is a zinc finger transcription factor essential for differentiation of immature hematopoietic cells (137). In addition, GATA2 regulates phagocytosis by alveolar macrophages (138), and GATA2 overexpression in alveolar macrophages increases phagocytic activity up to threefold (139).

Patients with GATA2 mutations present with numerous diagnoses and symptoms including MDS, AML, chronic myelomonocytic leukemia (CMML), severe viral, disseminated mycobacterial and invasive fungal infections, pulmonary arterial hypertension, warts, panniculitis, human papillomavirus (HPV)-positive tumors, EBV-positive tumors, venous thrombosis, lymphedema, sensorineural hearing loss, miscarriage, and hypothyroidism (140).

A very high rate of leukemia is seen in subjects with SCN. This is predominantly AML, but subjects may also suffer ALL, CML, and bi-phenotypic leukemia (141). Point mutations in the colony-stimulating factor 3 receptor (granulocyte) (GCSFR or CSF3R) gene are seen in bone marrow cells from most SCN patients who develop leukemia, suggesting that such mutations are highly predictive, although not essential, for malignant transformation in these subjects (142). CSF3R mutations are very specific for patients with SCN and are not found in patients with primary AML or other forms of chronic neutropenia requiring long-term treatment with G-CSF (142). These data emphasize the critical role of immune neutrophils in immunosurveillance of pre-cancerous cells.

## Defects in the Immune Response in Pid Patients Drives Malignancy

The data presented above suggest that a defective immune response in PID patients is associated with a greater incidence of cancer independent of whether the defect is primary or secondary. For example, lower serum Ig levels are associated with malignancies, highlighting the importance immunodeficiency in the predisposition to neoplasia (143). This risk is amplified by the enhanced susceptibility to viral infections in patients with PIDs (144). Defective immunosurveillance is undoubtedly a major factor in the risk of developing cancer, and this is seen most evidently in cells with a strong antigenic potential that have undergone viral induction. Supporting this concept is the fact that lymphoma, an immune system-related malignancy, is the most common cancer subtype in immunodeficient patients (12, 145). NHL and Hodgkin’s disease (HD) account for 48.6 and 10%, respectively, of the malignancies seen in patients with PIDs according to the Immunodeficiency Cancer Registry (ICR) database (124, 146). The overall risk for cancer developing in children with PID is estimated to range from 4 to 25% (12, 147). Finally, EBV infection in WAS patients induces lymphoma (135, 148), and the occurrence of lymphoproliferative disorders (LPDs) in patients with PID has been documented for nearly 40 years.

Mutations in the X-linked inhibitor of apoptosis (XIAP) are associated with a rare primary immunodeficiency. XIAP is an anti-apoptotic molecule but is also important in many other pathways, including control of innate immunity and in the negative regulation of inflammation (149). Loss of XIAP results in reduced NOD ligand-induced proinflammatory cytokine expression in both mouse and man (150–152). Furthermore, the ability of the pattern recognition receptor dectin-1 to recognize fungal β-glucan from fungi is modulated by XIAP (153). Under these conditions, XIAP regulated β-glucan-induced NF-κB and MAPK activation, cytokine production, and phagocytosis *via* ubiquitination of BCL10. The T-cell receptor (TCR) pathway activation of NF-κB also requires BCL10 and XIAP (151).

X-linked inhibitor of apoptosis deficiency is mostly reported in young boys who can be affected during the first few months of life, with symptoms being most severe in the youngest patients. The most frequent clinical manifestations are HLH (54%), recurrent splenomegaly (57%), and IBD (26%). Viral infection is often the trigger for HLH, with EBV (60% of cases) with cytomegalovirus (CMV) and human herpes virus 6 (HHV-6) being the most important, although symptoms may occur without an identified infectious agent (149).

These PIDs are a heterogeneous group of genetically determined disorders, which give rise to a number of diverse and variable LPDs. The susceptibility of developing LPD is associated with the type of PID presence, but accurate quantification of this risk is difficult since PIDs are rare. Analysis of case reports provides risk estimates of 0.7–15% (154). The extent to which primary immunodeficiency in man leads to increased cancer development is therefore unclear and generally relies on gene-targeted murine studies (155). For example, recombination-activating gene 2 (RAG2)-deficient mice that lack both T and B cells are more susceptible to spontaneous and carcinogen-induced carcinomas (156), while mice lacking γδT cells are highly susceptible to cutaneous carcinogenesis (157).

In contrast, interferon-α/β (IFN-α/β) and IFN-γ protect mice against both spontaneous and carcinogen-induced cancers (156, 158–160). Moreover, perforin, which is used by cytotoxic lymphocytes to kill target cells and is defective in FHL, is important for the surveillance of spontaneous lymphoma in mice (161).

Collectively, the human and mouse data reveal a consistent association between primary immunodeficiency and an increased incidence of cancers. Overall, two major mechanisms appear to be important for this link, prevention of infection and immunosurveillance and elimination of pre-malignant cells, and these are described in more detail below.

## Insufficient Functioning of the Immune System Allows Infection and Prevents Effective Immunosurveillance

The immune system confers protection against viral and bacterial pathogens and parasitic worms. Although evidence exists for the immune system targeting invading cancers, there are less data demonstrating the immunological eradication of pre-cancerous lesions in man and thereby preventing cancer (143, 162). For example, although organ transplant recipients who are treated with immunosuppressive drugs are more prone to cancer development (163, 164), the majority of posttransplantation lymphomas was associated with EBV infection (165). Thus, most of the lymphomas reported in PID patients are likely to be secondary events resulting from reduced antiviral immunity, rather than a direct effect of reduced antitumor immunity.

The HIV1 virus causes acquired immunodeficiency by selectively infecting and killing CD4+ T cells, and this is associated with an elevated risk of cancer. These cancers are generally linked to oncogenic viruses such as Kaposi sarcoma [caused by human herpes virus 8 (HHV8)], Hodgkin’s lymphoma and NHL (EBV), anal and cervical cancer (human papilloma virus), and liver cancer (hepatitis B and C viruses). Kaposi sarcoma, NHL, and cervical cancer are particularly frequent and are considered as acquired immunodeficiency syndrome (AIDS)-defining cancers (166). Blockade of the immune check-point molecules CTLA-4, PD-1, or PDL1 is of value in these cancers by potentiating the patient’s own immune response (167).

Elimination of pre-malignant cells can occur, and the importance of immune system in defending against cancers is exemplified by lymphocytes bearing the receptor killer cell lectin-like receptor subfamily K, member 1 (KLRK1), which are able to recognize and eliminate stressed pre-malignant cells (168). Self-proteins, such as MICA and MICB, are ligands for KLRK1 as are six different ULBP proteins that are poorly expressed in normal resting cells but whose cell surface expression is upregulated after treatment with DNA-damaging agents such as ionizing radiation and UV light (169, 170). Many freshly isolated lung, breast, kidney, ovary, prostate, colon, and liver carcinomas express MICA and MICB (171). The expression of these KLRK1 ligands is also induced by oncogenic growth factors acting through their receptors such as the epidermal growth factor receptor (EGFR) (172). The EGFR pathway is frequently dysregulated in human cancer, and EGFR activation may regulate the immunological visibility of stressed pre-malignant cells (169). KLRK1 knockout mice develop prostate adenocarcinomas and B cell lymphomas (173), and polymorphisms in the KLRK1 gene are associated with the susceptibility of developing liver and cervix cancers (174, 175).

In summary, the data from both man and mice suggest that the expression of stress-induced endogenous molecules associated with cell transformation may be used by the immune system to recognize and eliminate pre-malignant cells.

## Role of Infectious Agents in Driving Malignancy

The data to date suggest that the increase in viral infection seen in PIDs patients further enhanced the susceptibility to cancer, although the precise mechanisms for this remain unclear. The impact of infectious agents on cancer risk has been investigated in CVID patients. High HHV8 copy numbers are present in the malignant lymph node of CVID patients with granulomas (176). In addition, there is a 13-fold increase in CD8+ T cells specific for CMV-derived peptides in CVID patients compared with controls (177). Clinical observations indicate that an exaggerated T cell response to CMV may exacerbate enteropathy in CVID patients (147). Moreover, *Helicobacter pylori* infection and pernicious anemia are risk predictors for gastric cancer in CVID patients and in the general population (42).

Epstein–Barr virus is an important co-factor for the development of CVID and can affect the host, either primarily during an immunocompromised state or following reactivation during a disease flare (12, 178). The range of lymphoid tumors linked with EBV includes HD, angioimmunoblastic lymphadenopathy, lymphomatoid granulomatosis, some forms of HIV-associated lymphoma, and primary central nervous system lymphomas. It is paradoxical that a ubiquitous virus which causes a generally benign and self-limiting infective illness of childhood or early adult life may also be responsible for such aggressive tumors. Further studies are required to delineate the mechanism(s) of EBV and other viral infections on cancer susceptibility.

## Prognosis and Perspectives

We have described here how dysfunctions in the immune system of patients with PIDs can predispose patients to malignancies. Early treatment of the immunodeficiencies in some of these patients, before end organ damage has occurred, may reduce the risk of cancer, but clearly a greater understanding of the pathways responsible for this increased risk is needed. This may lead to the development of novel therapeutic agents that prevent cancer. In addition to the critical role in protection from infectious non-self-pathogens, the immune system is also important in modulating malignant self-challenges (cancer). Numerous immune cells are involved in cancer surveillance and prevention including those of the adaptive (T and B cells) and innate (NK and macrophages) immune systems, and all of these are defective to a greater or lesser extent in patients with PID. However, our knowledge of the precise mechanisms by which the immune system fights cancer, particularly in relation to infection by non-oncogenic viruses, remains rudimentary at best, although an inaccurate antitumor immune response may occur (179). There is no inherent difference in cancer treatment response in patients with PID compared to in non-immunodeficient patients (124). However, since patients with PIDs often have disseminated tumors that require systemic cytotoxic therapy which is poorly tolerated, there is an increased risk for infection and end-organ damage. New drugs are needed for these patients directed against the oncogenic pathways linked to each PID, but in the meantime, effective and early treatment of the immune defects present in each PID may reduce the associated risk of cancer.

The causative associations between immune factors and oncologic processes can be a pathological two-way street. For example, immunocompetent patients with cancer can develop immune suppression secondary to the cancer, from chemotherapy or from posttransplant immunosuppressive therapy (180). This is particularly evident for B-cell chronic lymphocytic leukemia and associated hypogammaglobulinemia. As in patients with PID, infection control is paramount. Immunoglobulin replacement therapy has been shown to reduce the frequency of bacterial infections in patients with chronic lymphocytic leukemia (181–184) and NHL (182), and this may impact upon the subsequent risk of malignancy.

**Figure 1:**
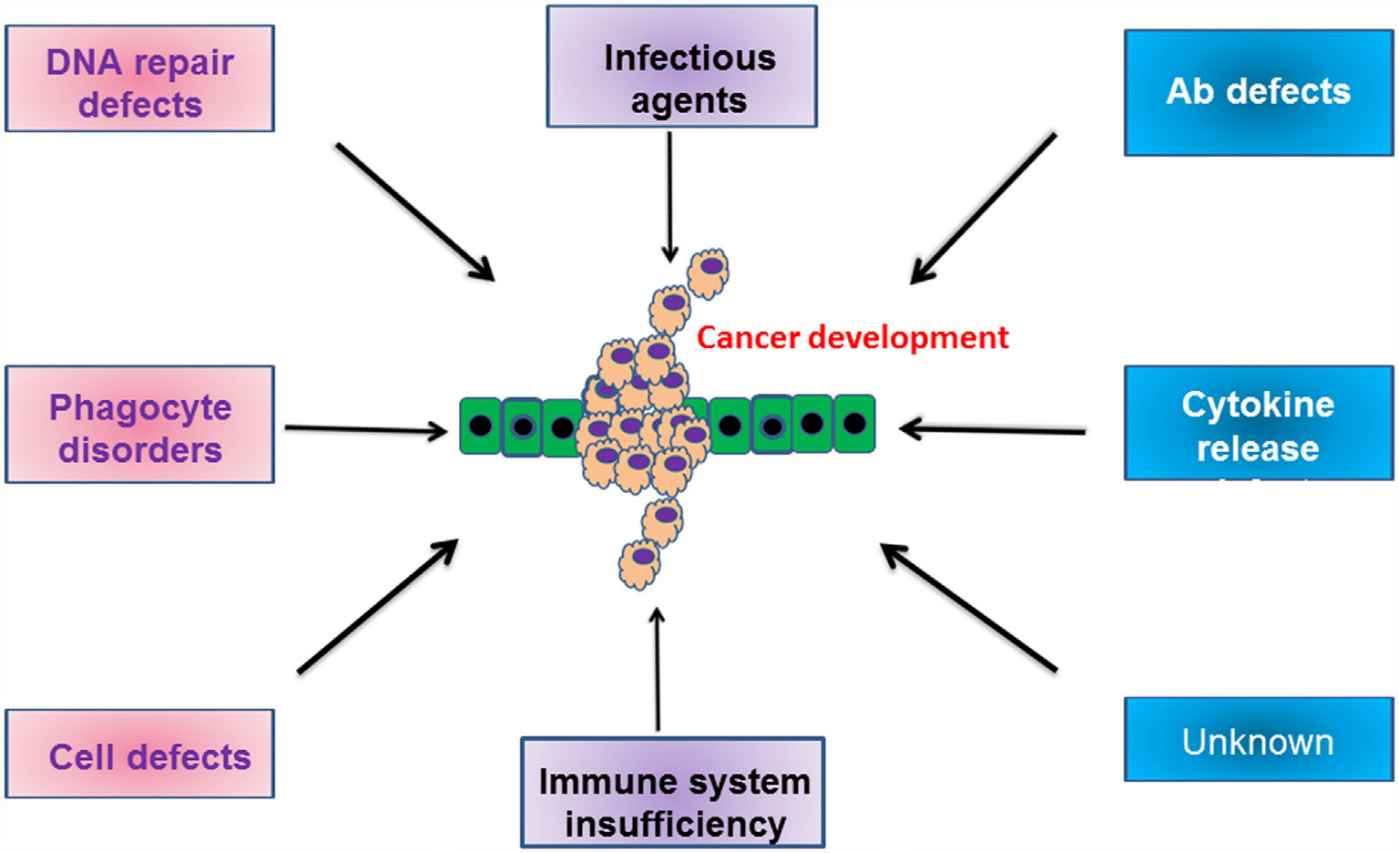
**Schematic cartoon indicating the various defects in humoral and cellular compartments, which drive the development of malignancies**. For more information, please refer to the text.

In conclusion, the increased incidence of cancer in patients with some types of PID is due to the inherent dysregulation of the immune response present and/or exposure to an infectious organism (Figure 1). It is only through greater understanding of how the specific mutations in each patient relate to and modify oncogenic pathways that we will prevent the development of these malignancies.

## Author Contributions

All authors contributed in writing and approval of the manuscript.

## Conflict of Interest Statement

The authors declare that the research was conducted in the absence of any commercial or financial relationships that could be construed as a potential conflict of interest.

## Abbreviations

ADA, adenosine deaminase; ADAM17, a disintegrin and metalloproteinase 17; AD-HIES, autosomal-dominant hyper-IgE syndrome; AICDA, activation-induced cytidine deaminase; AIDS, acquired immunodeficiency syndrome; AIRE, autoimmune regulator; APS, autoimmune polyendocrine syndrome; AR-HIES, autosomal recessive hyper-IgE syndrome; ATM, ataxia-telangiectasia mutated; BAFF-R, B-cell-activating factor receptor; BLNK, B-cell linker; BTK, B-cell tyrosine kinase; CD, cluster of differentiation; CD40L, CD40 ligand; CGD, chronic granulomatous disease; CIDs, combined immunodeficiencies; CMC, chronic mucocutaneous candidiasis; CMCD, chronic mucocutaneous candidiasis disease; CMV, cytomegalovirus; CN, congenital neutropenia; CTLs, cytotoxic T lymphocytes; CVID, common variable immunodeficiency; CYBA, cytochrome *b* alpha; CYBB, cytochrome *b* beta; DOCK8, dedicator of cytokinesis 8; EBV, Epstein–Barr virus; ELA, elastase; FHL, familial hemophagocytic lymphohistiocytosis; G6PC3, glucose-6-phosphatase, catalytic, 3; G-CSF, granulocyte colony-stimulating factor; GF, growth factor; HAX1, hematopoietic cell-specific Lyn substrate 1 (HCLS1)-associated protein X-1; HD, Hodgkin’s disease; HHV8, human herpes virus 8; HSE, herpes simplex encephalitis; ICOS, inducible co-stimulator; ICR, Immunodeficiency Cancer Registry; IFNGR, IFN γ receptor; IGHMM, Ig heavy chain mu; IGLL1, Ig light chain lambda; IL12R, IL-12 receptor; IL2RG, IL-2 receptor gamma; IL7R, IL-7 receptor; IRF4, interferon regulatory factor 4; JAK, janus-associated kinase; LPDs, lymphoproliferative disorders; MAPBP, mitogen-activated protein kinase-binding protein; MSMD, Mendelian susceptibility to mycobacterial disease; NBS, Nijmegen breakage syndrome; NCF, neutrophil cytosolic factor; NHL, non-Hodgkin lymphoma; NK, natural killer; PGM3, phosphoglucomutase 3; PIDs, primary immunodeficiencies; PRF, perforin; RAG, recombinase-activating gene; RMRP, RNA component of mitochondrial RNA processing endoribonuclease; RORγt, retinoic acid-related orphan receptor γt; SCN, severe congenital neutropenia; STAT, signal transducer and activator of transcription; STX11, syntaxin; TACI, transmembrane activator and CAML interactor (calcium modulator); Th17, IL-17 secreting T helper cell; TLR, toll-like receptor; TNFRSF13B, TNF receptor soluble factor; Tyk2, tyrosine kinase 2; UNG, uracil N-glycosylase; WAS, Wiskott–Aldrich syndrome; WASP, Wiskott–Aldrich syndrome protein; XIAP, X-linked inhibitor of apoptosis; XLA, X-linked agammaglobulinemia.
